# Biomimetic Electrochemical Sensors Based on Core-Shell Imprinted Polymers for Targeted Sunset Yellow Estimation in Environmental Samples

**DOI:** 10.3390/bios13040429

**Published:** 2023-03-28

**Authors:** Sumeet Malik, Adnan Khan, Hamayun Khan, Gul Rahman, Nauman Ali, Sabir Khan, Maria Del Pilar Taboada Sotomayor

**Affiliations:** 1 Institute of Chemical Sciences, University of Peshawar, Khyber Pakhtunkhwa 25120, Pakistan; 2 Department of Chemistry, Islamia College Peshawar, Khyber Pakhtunkhwa 25120, Pakistan; 3 Chemistry Institute, São Paulo State University (UNESP), Araraquara 14801-900, SP, Brazil; 4 Department of Natural Sciences, Mathematics and Statistics, Federal Rural University of the Semi-Arid, Mossoró 59625-900, RN, Brazil; 5 TecMARA, Faculty of Sciences, National University of Engineering, Av. Tupac Amaru 210, Rimac 15333, Lima, Peru

**Keywords:** adsorption, electrochemical sensors, molecularly imprinted polymers, precipitation polymerization, sunset yellow dye

## Abstract

Magnetic molecularly imprinted polymers (MMIPs) contain the predesigned specialized recognition capability that can be chosen to build credible functional materials, that are easy to handle and have a good degree of specificity. Hence, the given piece of work is intended to design a novel electrochemical sensor incorporating magnetite-based molecularly imprinted polymers. The building materials consisted of a cross-linker (EGDMA), reaction-initiator (AIBN), monomer (methylene succinic acid-MSA), and template molecule (Sunset Yellow-SY dye). MMIPs exhibited a diameter of 57 nm with an irregular shape due to the presence of cavities based on SEM analysis. XRD patterns exhibited crystallinity, as well as amorphous peaks that are attributed to polymeric and non-polymeric frameworks of MMIPs. The crystallite size of the MMIPs from XRD analysis was found to be 16.28 nm based on the Debye-Scherrer’s equation. Meanwhile, the FTIR bands showed the synthesis of MMIPs using monomer and methylene succinic acid. The sorption data at the optimized operating conditions (pH 2, sorbent dosage 3 mg, time 18 min) showed the highest sorption capacity of 40 mg/g. The obtained data best fitted to the Langmuir sorption isotherm and followed the pseudo-second-order kinetics. The magneto-sensors were applied for ultrasensitive, rapid, and simple sensing of SY dye. The electrochemical experiments were run at the operating condition range of (scan rate 10–50 mV/s, tads 0–120 s, pH 5–9, potential range 1–1.5 V for CV and 1–1.3 V for SWAdASV). The linear range of detection was set to 1.51 × 10^−6^ M to 1.51 × 10^−6^ M posing LOD and LOQ values of 8.6242 × 10^−5^ M and 0.0002874 M, respectively. The regression analysis value for the calibration was found to be 0.950. Additionally, high adsorption efficiency, selectivity, reusability, and strong structural stability of the magneto-sensors showed potential use for SY detection in real samples. These characteristics make MMIPs a viable electrochemical substrate for the detection of chemical contaminants in the environment and in health-related products.

## 1. Introduction

The rapid expansion of industrial activity, combined with improvements in science and technology, raises the standard of life and promotes global competition and sustainable economic growth [1]. This quick expansion comes with an escalating pollution problem and one of the world’s major issues is water pollution. Due to the increased use of water in the industrial, household, and agricultural sectors, water quality is deteriorating and drinking water supplies are declining [2,3]. Industrial runoff is full of harmful and infectious contaminants that have a negative impact on the ecology. Dyes are one of the most important types of toxins among the many industrial pollutants. These dyes are widely used by many industries, including the textile, rubber, automotive, cosmetics, pharmaceutical, printing, and photography sectors, to improve the aesthetic appearance of the products [4].

Dye-laden effluent from these enterprises is no longer beneficial and occasionally difficult to clean once it reaches the natural water system. Its complicated structure, synthetic composition, and resistant character make them more stable. Synthetic dyes are acknowledged as a type of noticeable pollution with a high color intensity even at extremely low concentrations. As a result, it needs to be removed before the effluent is released into the aqueous system [5]. One of these well-known food dyes is Sunset Yellow (SY) dye. A typical food color called Sunset Yellow (pyrazolone) is used in drinks, sweets, dairy products, and bread goods. Sunset Yellow (SY) dye is an azo dye in nature characterized by the presence of one or more azo bonds. The chemical name of SY dye is disodium 2-hydroxy-1-(4-sulfonatophenylazo) naphthalene-6-sulfonate [6]. The acceptable daily intake (ADI) defined by the FAO/WHO Joint Expert Committee on Food Additives (JECFA) in 2011 was 0–2 mg/kg. According to the European Food Safety Authority (EFSA), the ADI for SY should range from 1 to 2.5 mg/kg. Overuse of azo dyes can result in major health issues such as chromosomal abnormalities, allergic responses, and hyperactivity in youngsters. In this regard, it is strongly recommended to monitor SY dye presence and quantity [7,8]. Currently, a variety of analytical techniques, including optical emission spectroscopy [9], ultra-violet (UV) spectrophotometric method [10], micro-extraction [11], high-performance liquid chromatography (HPLC) method [12], and electrochemical sensors [13] are available for the detection of SY dye. Among these methods, electrochemical sensing has grabbed the attention of researchers on a massive scale, attributed to a more sensitive and selective nature than other analytical methods. However, procedures for separation and concentration may display a significant difficulty [14]. The design of MIPs demonstrates themselves as an alternate approach for highly targeted detection of analytes and offers a solution to this problem. Due to the existence of stereo chemically molded spots (or cavities/apertures) owing to template entities, the MIPs can choose a particular molecule, emulating a substrate-enzyme or an antibody-antigen system without being constrained by its properties (pH, temperature, and pressure). Available studies show many scientists’ and researchers’ attempts to improve the efficiency of MIPs and their biomimetic response toward the target analytes [15]. A combined approach of imprinted polymers and nanoparticles distinguishes them as having excellent efficiency and recognition sites. Using the co-precipitation approach, MIP polymerization takes place over nanoparticles (such as iron magnetite; Fe_3_O_4_) [16]. As opposed to the traditional-MIPs-based adsorption, this combination enables a more uniform format and size, as well as increased exposed sites. As a result, this particular adsorbent (Fe_3_O_4_@MIP) became suitable for sensor construction. The application of MIPs (Fe_3_O_4_@MIPs) onto the electrodes (electrochemical sensors) enhances their selectivity/specificity and makes them worthy of multiple applications [17]. The polymeric materials are non-conductive in nature, hence they are mixed with a conductive material, such as graphite to synthesize the MMIPs-based sensors [18,19]. Currently, a novel “core@shell-molecularly-imprinted-magnetic polymers-based sensors” (Fe_3_O_4_@MIPs) will be synthesized for the precise detection of SY dye in environmental samples. To our best knowledge, this MMIP-based electrochemical sensor will be the first one designed for the selective removal of SY dye.

## 2. Materials and Methods

### 2.1. Chemicals and Reagents

Sigma Aldrich provided with the ‘ethylene-glycol-dimethacrylate’ (EGDMA), ‘Sunset Yellow’ (SY), ‘2,2-azobisisobutyronitrile’ (AIBN), ‘methylene succinic acid’, ‘ethanol’, ‘iron sulfate, and iron chloride (USA). Graphite powder was purchased from Fluka Powder. It has a high level of electric conductivity. Using the Henderson-Hasselbalch equation, phosphate buffer solutions of various pH values were prepared. Monobasic and dibasic potassium phosphate at varying concentrations were utilized throughout the experiment.

### 2.2. Synthesis of MMIPs and MNIPs

The magnetite nanoparticles were synthesized through the co-precipitation method [13]. The modification of the magnetic nanoparticles was performed using TEOS. In this step, magnetite NPs (300 mg) were mixed with ethanol (40 mL) and deionized water (4 mL). The mixture was left to sonicate for 15 min followed by the addition of tetra-ethoxyorthosilicate (2 mL) and ammonium hydroxide (5 mL). The mixture was left to react for 10 h on a magnetic stirrer. After the completion of this step, the product was washed, dried, and stored. Next, TEOS-Fe_3_O_4_ (250 mg) were further modified by adding 3-metacriloxipropiltrimetoxissilane (5 mL) and anhydrous toluene (50 mL). These combined reactants were again stirred for 10 h under nitrogen protection. After reaction completion, the product was washed, dried, and stored for further usage.

The precipitation polymerization method [19] was used to synthesize magnetic MIPs (Fe_3_O_4_@MIPs). A reaction mixture containing 0.2 mmol SY dye and 0.8 mmol methylene succinic acid in 30 mL ethanol was agitated to initiate the monomer-template reaction. Next, 200 mg of modified magnetic nanoparticles were added to the mixture and stirred for 3 h further. Afterward, 4.0 mmol of EGDMA and 0.05 mmol of AIBN was added to the mixture. The reaction was allowed to run for 12 h at 60 °C under nitrogen flow. 

After the completion of polymerization, MMIPs were washed using an eluent to remove the template (SY). The eluent consisted of C_2_H_5_OH:CH_3_COOH mixture (composition~9:1). The acidic component of the eluent mixture causes the efficient expulsion of template molecule. The “non-imprinted polymers” were also designed through the same protocol except for the addition of template molecule.

### 2.3. Characterization

Using scanning electron microscopy (SEM) from JEOL [JSM-5910-Akishima, Tokyo, JAPAN], the morphological characteristics of the MMIPs and MNIPs were identified. With Image J, it was possible to determine the average particle size. The MMIPs were validated through “Fourier-transform-infrared-spectroscopy” (400–4000 cm^−1^) ATR-equipment (Thermo Electron Corporation, Waltham, MA, USA). Energy-dispersive X-ray spectroscopy (EDX) was also used for elemental analysis (Oxford Instruments-INC-200, Abingdon, Oxfordshire, England, UK). Crystalline phase of the product was confirmed via ‘X-ray diffraction’ [JEOL-JDX-9C-XRD- Akishima, Tokyo, JAPAN-spectrometer].

### 2.4. Electrochemical Assay

Employing a potentiostat, the study of “MMIP-sensor” was examined (Gamry Interface 1010B, GAMRAY Instruments, Warminster, PA, USA). The electrochemical experiments were carried out in a three-electrode electrochemical cell. The reference electrode was made of silver/silver chloride (Ag/AgCl). The counter electrode was a platinum plate. Utilizing carbon paste (intermingled with MMIPs) within a “silver-ring” with Teflon inserted, the functional electrode was constructed. The working sensor electrode for the “non-imprinted-polymer” (MNIPs) was also designed via similar protocol. A phosphate buffer solution (0.1 mol/L, pH: 7.8) was used for running the electrochemical measurements.

### 2.5. Electrochemical Sensor Design and Manufacturing

The working electrode in this study was composed of a Teflon-based electrode (1 mm depth, 1.5 mm interior diameter) filled with “carbon-paste”. To develop a homogenous paste, 85 mg of “graphite-powder” and 15 mg of MMIPs were combined (with a few drops of water). It was ensured that the graphite powder was well mixed with the MMIPs. At room temperature, the paste was allowed to set for 24 h. The mixture was then thickened with paraffin oil (1 mL). A platinum disc was used to apply the electrical contact while the “Teflon-hollow-chamber” was filled with the paste.

### 2.6. Sorption/Binding Assay

For evaluating the binding performance of polymers (both imprinted and non-imprinted), a sorption assay had been carried out at the optimized experimental conditions. For sorption procedure, 1–12 mg of sorbent (MMIP/MNIP) was added to 10–100 ppm of analyte (SY) solution with a volume of 0.01 L at pH 2–9 and shaken on a homogenizer for 3–18 min. After the completion of the sorption process, the polymers were separated from the mixture via centrifugation. Furthermore, the supernatant was analyzed using a UV-vis spectrophotometer at λmax of 490 nm. The sorbed amount of analyte was calculated using Equation (1).

(1) $$Q_{e}=\frac{C_{i}-C_{f}}{m}\times V.$$

where “*Q_e_*” stands for the sorption capacity (mg/g), “*C_i_*”and “*C_f_*” presents the initial and final dye concentration respectively, “*m*” is for the mass of sorbent (g) and “*V*” is the volume of dye solution (L) [20].

## 3. Results

### 3.1. Scanning Electron Microscopy

**Figure 1:**
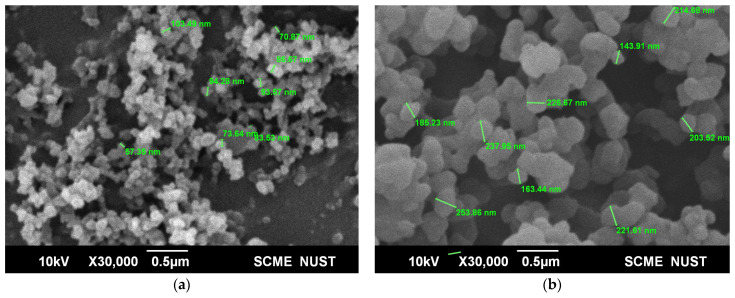
SEM images of (**a**) MMIPs (**b**) MNIPs.

Scanning electron microscopy was used to determine the surface morphology of the magnetic molecularly imprinted polymers and magnetic molecularly non-imprinted polymers (Figure 1a,b). As clearly seen from the SEM images, the MMIPs showed a smaller size with somewhat rough morphology and irregular shapes. This roughness can be attributed to the presence of cavities on its surface, while the magnetic non-imprinted particles had a more spherical morphology based on the fact that no cavities are present on their surface. When compared to MNIPs (average diameter 226 nm), the average diameter of MMIPs (57 nm) generated (through image-J) offered greater locale reachability, reduced bulk transferal reluctance, as well as targeted morphology toward analyte [21,22].

### 3.2. Fourier-Transform Infrared Spectroscopy

**Figure 2:**
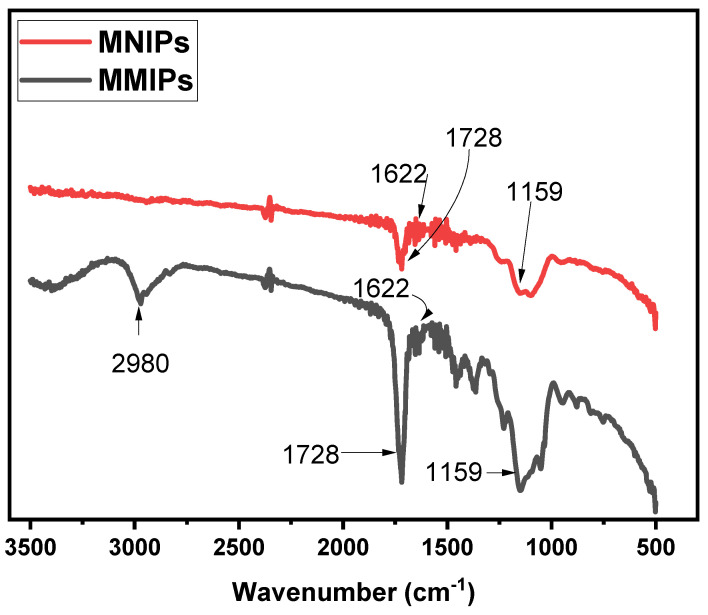
FTIR spectra obtained to MMIPs and MNIPs.

The structural analysis of both the MMIPs and MNIPs was performed using Fourier-Transform-Infra-red Spectroscopy. The results obtained for MMIPs and MNIPs are presented in Figure 2. In the spectrum of MMIPs, a sharp band observed at 1729 cm^−1^ referred to the C = O carbonyl stretch [23]. A band observed at 2980 cm^−1^ showed C-H bonds of EGDMA. The bands present at 1159 cm^−1^ and 1622 cm^−1^ corresponded to the C-O stretching and vinyl groups of methylene succinic acid, respectively [24,25,26]. A sharp band present at 532 cm^−1^ showed the Fe−O absorption band of Fe_3_O_4_ [27]. A comparison of the spectrum of MMIPs with that of MNIPs showed almost similar bands were observed for both, confirming successful synthesis.

### 3.3. Elemental Study Using Energy-Dispersive X-rays

**Figure 3:**
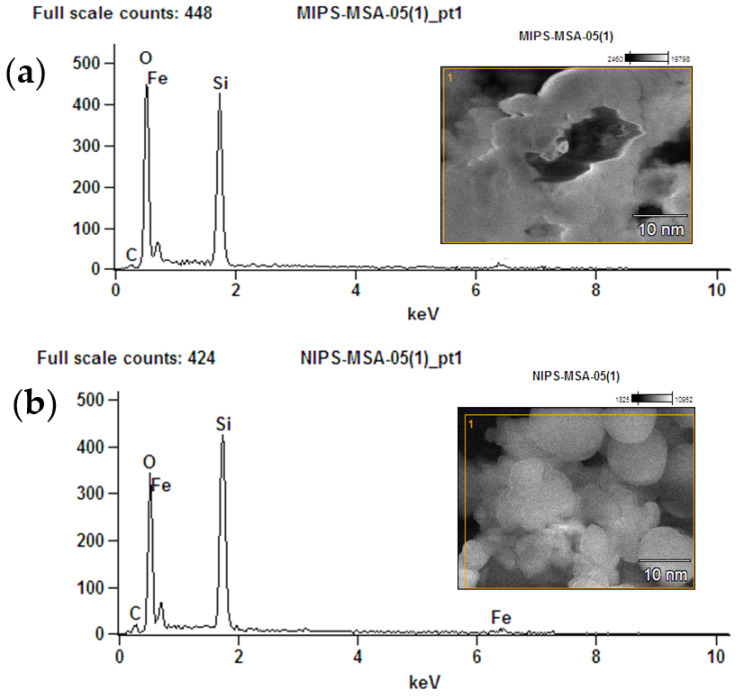
EDX analysis (**a**) MMIPs and (**b**) MNIPs.

The element’s investigations for MMIPs as well as MNIPs were carried out using EDX analysis. Elements Si, Fe, C, and O were observed in EDX spectrum of MMIPs (Figure 3a,b). The MSA, EGDMA, and SY dye are responsible for the O and C peak. The Fe peak confirmed the magnetite formation. While the silicon presence confirmed the modification of the polymers.

### 3.4. XRD Analysis

**Figure 4:**
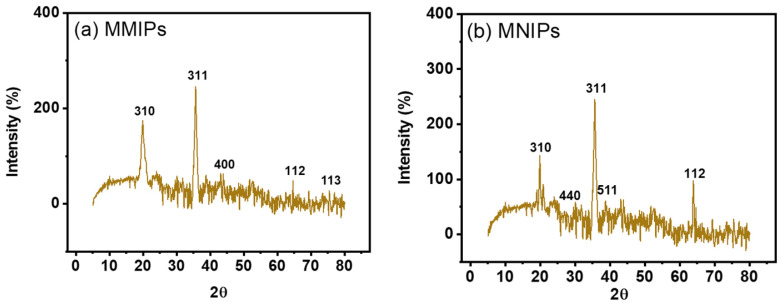
XRD spectra of (**a**) MMIPs and (**b**) MNIPs.

The products’ crystalline makeup was assessed using X-ray diffraction (XRD) examination. Figure 4b,c displayed the XRD-diffractogram of MMIPs & MNIPs. These patterns demonstrated that the product contained both crystalline and amorphous structures. The organic polymeric structures seen in the MMIPs and MNIPs have fused peaks. The Fe and Si impart crystallinity to the MMIPs and MNIPs as shown by the strong peak found in the diffractogram [28,29]. The magnetite found on the polymeric framework is represented by the peaks found at 35.6, 19.9, 64.6, 43.1, and 75.4. According to the earlier study, the corresponding planes at 311, 310, 112, 400, and 113 represented the spinel (cubic) crystalline structure of magnetite. Silica’s presence causes the peaks found at 38.7, 27.8, 49.9, and 66.9 to correspond to the planes 511, 440, 012, and 112 due to its crystalline-phase (JCPDS no. 19-0629) [30]. Deploying “Debye-Sherrer’s equation” (Equation (2)), the particle size/crystallite size was computed.

(2) $$D=\frac{K\lambda }{\beta cos\theta }$$

where “*D*” denotes ‘particle’s size’, ‘*K*’ denotes a constant, ‘*λ*’ denotes the incoming “X-ray-wavelength”, “*β*” denotes “full-width-half-maxima”, while “*β*” is the ‘diffracted-Bragg’s-angle’ [31]. While MNIPs’ particle’s size was 16.28 nm, MMIPs’ particle’s size was 15.52 nm considering the highest peak obtained in XRD spectrum. 

### 3.5. Binding and Sorption Studies

**Figure 5:**
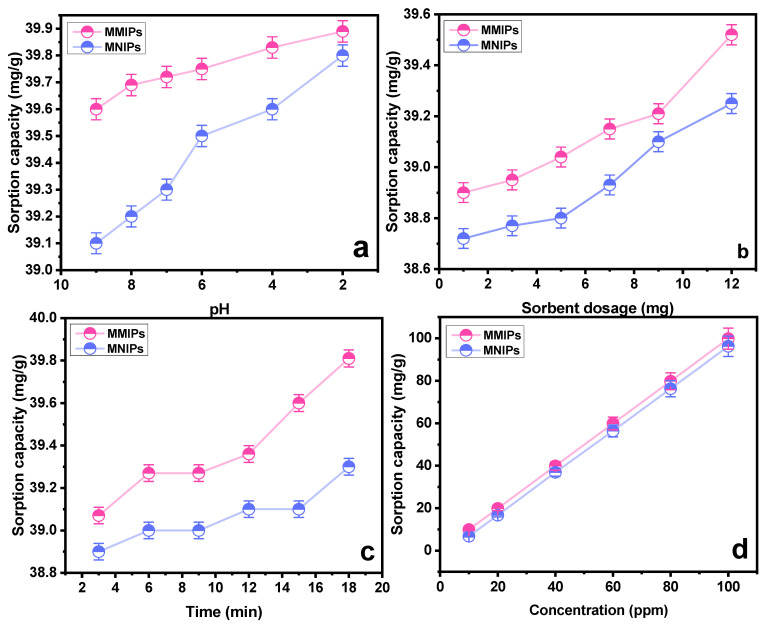
Effect of (**a**) pH on sorption of SY dye onto MMIPs and MNIPs (**b**) sorbent dosage on sorption of SY dye onto MMIPs and MNIPs (**c**) time on sorption of SY dye onto MMIPs and MNIPs (**d**) concentration on sorption of SY dye onto MMIPs and MNIPs.

To evaluate the sorption capacity of the polymers, and confirm their selectivity, binding studies were performed. The obtained results are portrayed in the Figure 5a–d. The effect of pH on the sorption capacity was studied by keeping other parameters constant. As obvious from the findings, the MMIPs showed a higher sorption capacity than the associated MNIPs. This relative difference is attributed to the presence of template-specific cavities in MMIPs, which are lacking in the MNIPs [32]. It is depicted from the results that the sorption reached its maximum at pH 2. As the pH rises towards more basic, the negative charges on the sorbent surface increases. The carboxylic group of methylene succinic acid is converted into anion carboxylate. As the negative charge of sorbent increases, it exerts a repelling force toward the anionic SY dye. The dissociated SY dye (sulfonate ions D−SO3−) at acidic pH garners greater electrostatic interaction towards the positively charged sorbent at acidic conditions [33]. Another group of researchers [34] also studied the effect of pH on the sorption of sunset yellow dye using a molecularly imprinted polymer. It was observed that a maximum interaction between the dye and sorbent was achieved at pH 5, which is an acidic pH. The protonation of dye in the aqueous medium affects the sorption process substantially. The MMIPs and MNIP (sorbents) tend to acquire a surface charge when interacting with molecules of water, which causes charge effects to occur adjacent to the nanoparticles. At pH 5, the dye gets dissociated, producing sulfonate anions which interact with the sorbents through electrostatic interaction.

The second important parameter affecting the sorption process is the sorbent amount. Multiple runs with a varying amount of sorbent were performed (1−12 mg). Figure 5b showed the findings of MMIPs and MNIPs at varying dosages with respect to the sorption capacity. It can be concluded that an increase in sorption capacity is associated with the availability of a greater number of available binding sites. Similar results were obtained by a group of scientists [35] who synthesized a nanocomposite of GO/Clay/Fe_3_O_4_@PDA MIP. Nanocomposite was used for the removal of the diazinon pesticide with an efficiency of 99% at an optimized sorbent dosage of 1.24 g.

**Figure 6:**
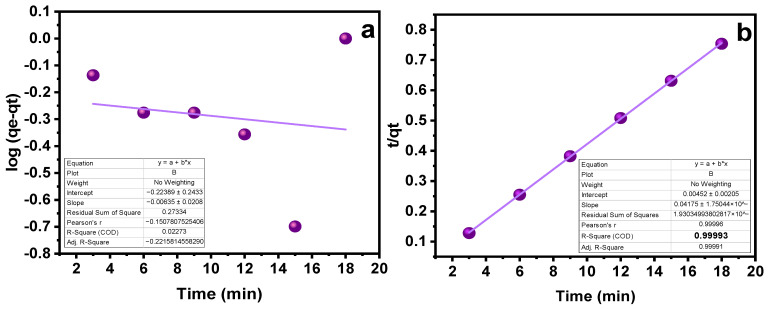
Kinetic studies (**a**) Pseudo−1st−order kinetics (**b**) Pseudo−2nd−order kinetics.

The impact of contact time on the SY’s sorption capability was assessed at different intervals of time. As indicated in Figure 5c, a time range of 3 to 18 min was chosen, with an increase of three minutes for each run. As the contact period was prolonged at first, the sorption capacity grew as well, but over time, this trend stopped, indicating that the MMIPs and MNIPs had reached their saturation point [36]. A maximal sorption capacity was attained at 18 min, as obvious from Figure 5c. The kinetic studies provide details on the rate−controlling phase aid in understanding the sorption process. To do this, the data were subjected to the application of pseudo-first-order kinetic and pseudo-second-order kinetic models (Table 1). The results demonstrated that the pseudo-second-order kinetic model best fitted the sorption process with R^2^ = 0.999 (Figure 6a,b). It shows that the binding is directly correlated to the square number of the vacant spots. The mechanism followed in the sorption process is the electrostatic or ionic interaction. The sorption process is shown to take place in two phases via the pseudo-second-order kinetic model. Firstly, the SY diffuses externally onto MMIPs followed by the development of electrostatic interactions [37]. Hence, a favorable sorption is observed in which the rate of reaction depends on both of the reactants.

**Figure 7:**
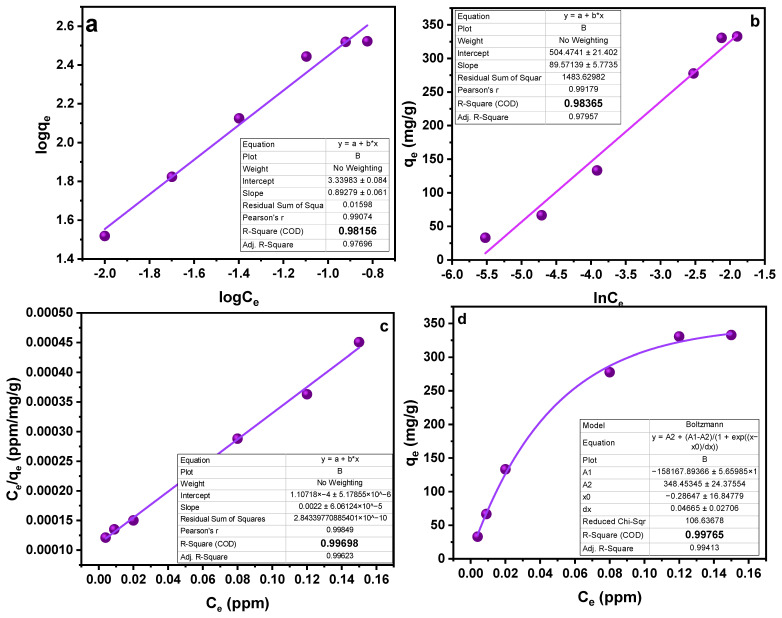
Isotherm studies (**a**) Freundlich Isotherm (**b**) Temkin Isotherm (**c**) Langmuir isotherm (linear) (**d**) Langmuir isotherm.

Another factor affecting the sorption process is the concentration of the analyte. To study the effect of concentration on the sorption process, different experiments were run in the range of 10–100 ppm concentration. A general idea that can be acquired from the trend obtained in Figure 5 is that sorption capacity increases with the increasing concentration [38]. Utilizing “Freundlich”, “Langmuir”, and “Temkin” sorption isotherms, the collected data were examined (Table 2). Based on high values of the linear regression coefficient (R^2^), as shown in Table 2, the data are better suited to the Langmuir adsorption model. In light of the fact that this model works well for a very homogenous surface, it is reasonable to assume a homogenous surface with a monolayer physisorption phenomenon (Figure 7). The RL values in the reported work were in the range of 1.2–3.5, higher than zero, demonstrating favorable sorption. Furthermore, advantageous sorption at a lower concentration was indicated by greater RL values at lower concentrations [39,40]. The results obtained as a result of isotherm study showed a feasible and satisfactory sorption ability of the MMIPs.

### 3.6. Electrochemical Experiments

**Figure 8:**
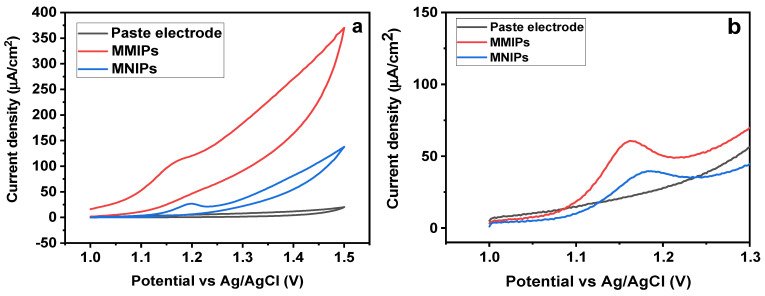
(**a**) CV for MMIPs, MNIPs, and Blank (**b**) SWAdASV for MMIPs, MNIPs, and Blank.

First, it was established that preconcentration had no discernible impact on the sensor’s response. The subsequent trials did not make use of this analytical parameter. Then, in the presence of SY dye (1.5 × 10^−3^ mol/L), comparison studies using carbon paste electrode (CPE), Fe_3_O_4_@MIP/CPE (MMIPs), and Fe_3_O_4_@NIP/CPE (MNIPs) electrodes were carried out [41,42]. The electrochemical profiles of the aforementioned electrodes, as calculated by “square−wave−adsorptive−anodic−stripping−voltammetry” (SWAdASV) technique, are shown in Figure 8a. The electrolyte used is a “0.1 mol/L phosphate buffer” (pH 7.0) and the analyte exhibits a distinctive “anodic-current-peak” at around 1.16 V (E vs. Ag/AgCl). The anodic peak currents of different electrodes are obtained as 0.44 (CPE), 0.68 ± 0.01 (MNIP/CPE), and 1.05 ± 0.01 (MMIP−GO/CPE). This shows that the sensor upgraded with MMIP/CPE gave the best electrochemical response (triplicate trials), that is 6.0 and 4.0 times greater than the response generated by NIP/CPE and CPE electrodes, respectively. By performing cyclic voltammetry, the efficiency of the electrode was analyzed (Figure 8b) [43]. In contrast to MNIPs and the blank solution, a more pronounced and stronger oxidation peak was seen for MMIPs, demonstrating a greater susceptibility to SY. The preconcentration of the dye onto the magneto-surface sensor owing to the selective cavities is responsible for the high oxidation peak current of MMIPs. The electrode’s excellent performance with MMIP must be attributed to the analyte’s selective sorption, which the control polymer (MNIP) did not exhibit [44].

**Figure 9:**
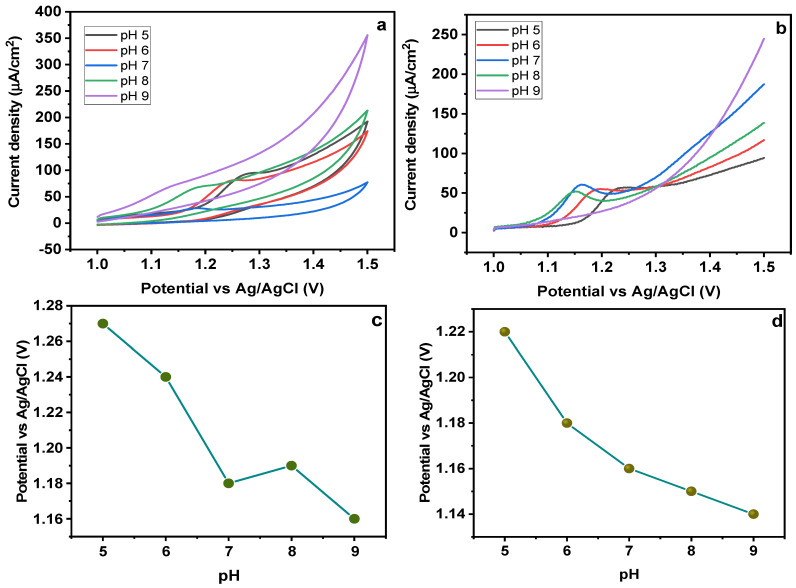
Effect of pH on (**a**) CV and (**b**) SWAdASV for Fe_3_O_4_@MIP/CPE sensors (**c**) pH vs peak potential for CV (**d**) pH vs peak potential for SWAdASV. (scan rate = 50 mV/s).

To evaluate the effect of pH on the electrochemical response of MMIP-based electrode, a series of experiments were performed with varying pH in the range of 5–9. A CV response for different pH solutions is given in Figure 9a. It is obvious from the results that with an increasing pH, a cathodic shift in the peak potential is observed. With regard to the oxidation peak, the peak potential (Epa) changed linearly with pH, demonstrating the ease of dye oxidation at comparatively lower applied potential at high pH values [45]. Additionally, the sharpest peak was attained at pH 7 with lowest peak potential and larger peak area as compared to the others, showing that pH = 7 can be an optimum value for sunset yellow electrochemical oxidation reversibility. Therefore, a pH of 7 was chosen for further investigation. Figure 9b shows the square wave adsorptive anodic stripping voltammetry (SWAdASV) response of the electrode at varying pH (5–9). Depending on the maximum peak current recorded during SWAdASV studies, the optimal pH was selected. The peak’s height was relocated to lesser potential values, when the solutions’ pH rose from 5 to 9. At pH 5–9, the anionic form of SY dye is predominating, hence, it builds strong electrostatic interactions with the cavities. It was seen that the highest anodic current value was attained at pH 7, so this was considered optimum for further analysis [46,47]. A graph between pH and peak potential has also been given (Figure 9c,d), showing a shift of peak potential with changing pH. A group of researchers also studied the influence of pH on the oxidation of Sunset Yellow dye rGO-g-CN/ZnO-AuNPs-based material. The CV experiments were conducted at 50 mV/s. The results showed that a maximal peak current was obtained at pH 7. Their findings showed that the current values rose to a maximum at pH 7, then to a minimum at pH 11. This can be because SY deprotonation occurs at higher pH.

**Figure 10:**
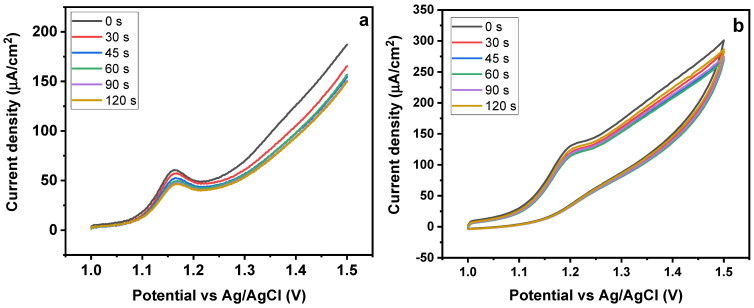
Effect of time on (**a**) CV and (**b**) SWAdASV for Fe_3_O_4_@MIP/CPE sensors (pH 7, scan rate 50 mV/s).

The electrochemical process includes the sorption of SY onto MMIP electrode surfaces, hence, it is important to look at the effects of both the accumulation time and the potential value used during the accumulation stage [48]. Additionally, the impact of pre-concentration time was assessed, utilizing a continuous range of sorption-time of 30–120 s (Figure 10a,b). The resulting SWAdASV profile revealed that the peak current intensity was initially high but, after certain time, there was no significant change in the intensity. It shows that, initially, the surface of the sensor exhibited high sorption capacity, leading to a good response. Once the surface of the electrode is completely covered with the SY molecules, there is a declining pattern in the current values as no more empty sites are available for further sorption (Figure 10a). A related trend was obtained in the case of CV obtained for SY detection for 0–120 s Figure 10b. Sunset yellow’s oxidation peak currents were not enhanced by longer accumulation times, indicating that their volume is likely to be limited [49].

**Figure 11:**
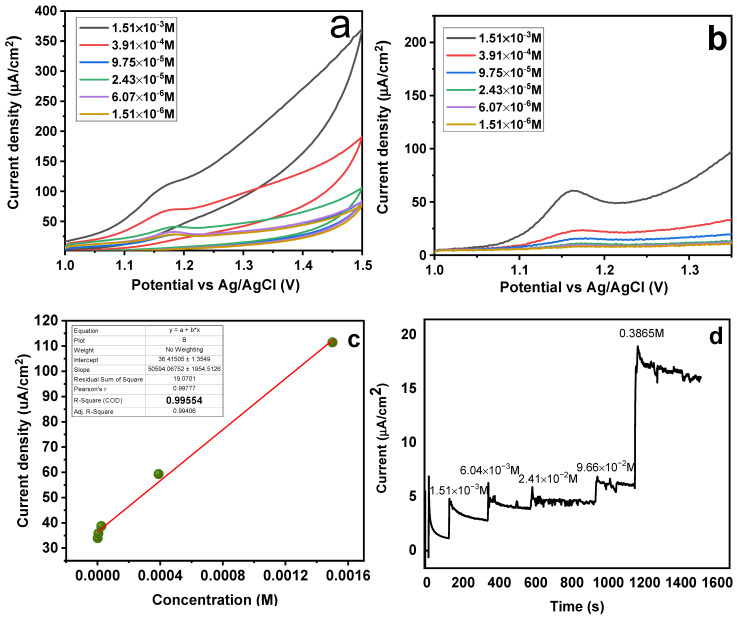
Effect of concentration on (**a**) CV and (**b**) SWAdASV for Fe_3_O_4_@MIP/CPE sensors (**c**) Calibration curve (**d**) Chronoamperometric response of magneto-sensors (potential 1.18 V).

It was examined how SY concentrations in the range of 1.51 × 10^−6^ to 1.51 × 10^−3^ mol L^−1^ affected the performance of MMIP-sensors (Figure 11a,b). According to the data, the analyte’s high concentration caused the sensor’s peak and current intensities to rise. These findings may be explained by the magneto-sensors’ high sensitivity to the analyte; hence, the sensor’s response increased with analyte concentration [50]. It can be observed from Figure 11a,b that a maximum response for both CV and SWAdASV was observed at the highest concentration of 1.51 × 10^−3^ mol L^−1^. With increasing concentration, the peak potential showed a slight cathodic shift, because concentrated analytes are easily oxidized owing to less solution resistance [51]. Different SY concentrations were employed in this investigation, ranging from 0.00156−0.39936 M (Figure 11d) with an increment of four times for each run. The data was plotted as a function of time (s) and current density (µA/cm^2^) by applying a potential of 1.18 V. The purpose of performing the chroamperometric tests was to confirm the diffusion of analyte (SY dye) onto the sensor surface, following a diffusion-controlled process (Figure 11 c,d). A “calibration curve” was made by plotting the SY concentration (M) versus the current density (µA/cm^2^). The resultant value of regression analysis was discovered to be 0.95, demonstrating the magneto-sensors’ strong linear behavior and validity within the given range [52]. The slopes were used to determine the LOD and LOQ respectively, which stands for the standard deviation of three blank runs. LOD and LOQ values were determined to be 8.6242 × 10^−5^ M and 0.0002874 M, using the formulas 3∂/slope and 10∂/slope, respectively.

**Figure 12:**
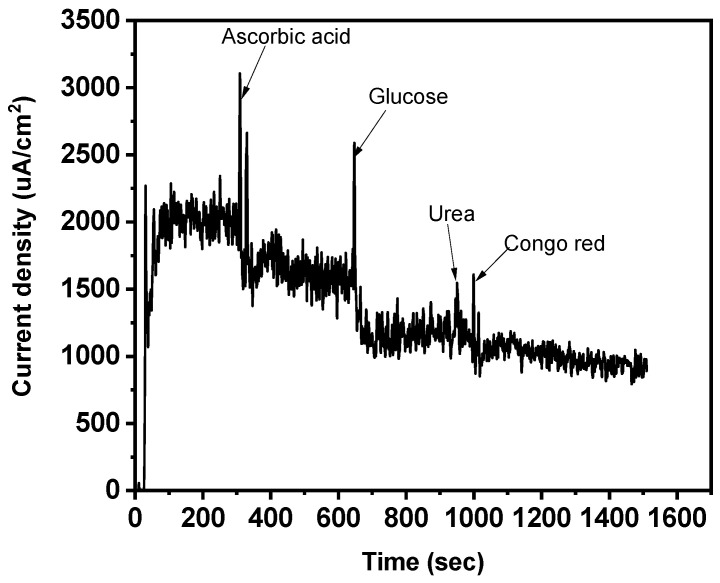
Effect of scan rate on CV and interfering species (potential 1.18 V).

An interference analysis was also carried out to assess the magneto-sensors’ strong specificity and selectivity toward the template (SY). Congo red dye, glucose, urea, and ascorbic acid were chosen and thought to be the main interfering species, especially working with realistic materials (Figure 12) [53,54]. The findings demonstrate that no discernible interaction of the interferents was noted, because the current intensity barely changed. This demonstrated how highly selective the magneto-sensor was for SY dye.

The proposed magneto-sensor was used to quantify SY-dye in “environmental samples” under optimal circumstances, as illustrated in Table 3. The findings demonstrated the magneto-sensor’s efficiency for the detection of SY as a sharp oxidation peak was obtained. The efficiency of magneto-sensor for the real sample was calculated using the spiking method. Three different concentrations of 6.07 × 10^−6^ M, 2.43 × 10^−5^ M, and 3.91 × 10^−4^ were spiked without any pre-treatment. The percent recovery obtained for the constructed sensor in industrial samples was 98 to 103%. The results are tabulated in Table 3. In such a way, the green sensing technology usage might help in the identification of these analyte(s) in many environmental samples and production procedures without any interference [55,56,57,58,59]. The comparison of current work with the already reported literature is shown in Table 4.

## 4. Conclusions

Electrochemical sensors have emerged as a promising tool for the selective detection of analytes. Based on these findings, MMIP-based electrochemical sensor was developed for SY dye quantification. A comparison of the detection capability of an imprinted sensor with that of a non-imprinted sensor was carried out. It was observed that better efficiency was portrayed by the imprinted sensor as compared to non-imprinted sensor. The designed MMIP-based electrochemical sensors have emerged as novel tools intended to perform the sensing and subsequent removal of the SY dye.

Furthermore, the sensor displayed significant electrochemical sensitivity and exceptional mechanical stability in terms of real samples. In summary, the electrochemical sensors showed a satisfactory response with LOD and LOQ values of 8.6242 × 10^−5^ M and 0.0002874 M, respectively. This confirms the broad horizon of MMIP-based sensors in a vast variety of food, medicine, and environmental materials. 

## Figures and Tables

**Table 1:** Kinetic Parameters for Pseudo−1st−order kinetics and Pseudo−2nd−order kinetics.

Pseudo−1st−Order Kinetics	
**Parameters**	**Values**
K	0.0146
*Q_e_*	0.5973
R^2^	0.02
**Pseudo−2nd−Order Kinetics**	
*Q_e_*	23.95
K_2_	12,6371.68
R^2^	0.999

**Table 2:** Isotherm parameters for Freundlich isotherm, Langmuir isotherm, and Temkin isotherm.

Freundlich Isotherm	
**Parameters**	**Values Obtained**
N	1.1201 L/g
K_F_	2182.72 mg/g
R^2^	0.981
**Langmuir Isotherm**	
**Parameters**	**Values Obtained**
K_L_	0.9032 L/g
a_L_	0.0019 L/mg
q_o_	475.3 mg/g
R^2^	0.996
**Temkin Isotherm**	
**Parameters**	**Values Obtained**
B_T_	−47.21 mg/g
A_T_	0.8063 L/g
R^2^	0.983

**Table 3:** Recovery values obtained in the application of the sensor in industrial samples.

Samples	Added/M	Found/M	Recovery (%)
Industrial sample 1	6.07 × 10^−6^	(5.94 ± 0.03) × 10^−6^	98
Industrial sample 2	2.43 × 10^−5^	(2.5 ± 0.06) × 10^−5^	103
Industrial sample 3	3.91 × 10^−4^	(3.98 ± 0.04) × 10^−4^	102

*Percent recovery* = (obtained/Added) × 100 Standard deviation for triplicates analysis.

**Table 4:** Comparative study of analytical parameters obtained with different molecularly imprinted polymers.

Material	Analyte	LOD	Reference
Magnetic molecularly imprinted polymers	Folate	1.0 × 10^−7^ mol L^−1^	[60]
Magnetic molecularly imprinted polymers	Sunset Yellow	0.00413 mol L^−1^	[13]
Magnetic molecularly imprinted polymers	Glutathione	0.07 μmol L^−1^	[61]
Magnetic molecularly imprinted polymers	Methyl Green dye	1.0 × 10^−8^ mol L^−1^	[14]
Magnetic molecularly imprinted polymers	Ametryn	25 nmol L^−1^	[62]
Polypyrrole-based molecularly imprinted polymer (MIP)	Methotrexate	2.7 × 10^−9^ mol L^−1^	[63]
Magnetic molecularly imprinted polymers	Tartrazine	0.303 μmol/L	[64]
rGO-g-CN/ZnO-AuNPs	Sunset Yellow Dye	1.34 nmol L^−1^	[65]
Magnetic molecularly imprinted polymers	Sunset Yellow Dye	8.6242 × 10^−5^ mol L^−1^	Present work

## Data Availability

Not applicable.
